# Mechanism of Dihydromyricetin on Inflammatory Diseases

**DOI:** 10.3389/fphar.2021.794563

**Published:** 2022-01-18

**Authors:** Yang Sun, Shasha Liu, Songwei Yang, Chen Chen, Yantao Yang, Meiyu Lin, Chao Liu, Wenmao Wang, Xudong Zhou, Qidi Ai, Wei Wang, Naihong Chen

**Affiliations:** ^1^ Hunan Engineering Technology Center of Standardization and Function of Chinese Herbal Decoction Pieces and College of Pharmacy, Hunan University of Chinese Medicine, Changsha, China; ^2^ Pharmacy Department, Xiangtan Central Hospital, Xiangtan, China; ^3^ Department of Pharmacy, The First Hospital of Lanzhou University, Lanzhou, China; ^4^ Zhangjiajie Meicha Technology Research Center, Hunan Qiankun Biotechnology Co., Ltd, Zhangjiajie, China; ^5^ TCM and Ethnomedicine Innovation and Development International Laboratory, Innovative Materia Medica Research Institute, School of Pharmacy, Hunan University of Chinese Medicine, Changsha, China; ^6^ State Key Laboratory of Bioactive Substances and Functions of Natural Medicines, Institute of Materia Medica and Neuroscience Center, Chinese Academy of Medical Sciences and Peking Union Medical College, Beijing, China

**Keywords:** inflammation, neuroinflammation, DHM, ampelopsisgrossedentata, mechanism

## Abstract

Inflammation plays a crucial role in a variety of diseases, including diabetes, arthritis, asthma, Alzheimer’s disease (AD), acute cerebral stroke, cancer, hypertension, and myocardial ischemia. Therefore, we need to solve the problem urgently for the study of inflammation-related diseases. Dihydromyricetin (DHM) is a flavonoid mainly derived from Nekemias grossedentata (Hand.-Mazz.) J.Wen and Z.L.Nie (*N.grossedentata*). DHM possesses many pharmacological effects, including anti-inflammatory (NLRP-3, NF-κB, cytokines, and neuroinflammation), antioxidant, improving mitochondrial dysfunction, and regulating autophagy and so on. In this review, we consulted the studies in the recent 20 years and summarized the mechanism of DHM in inflammation-related diseases. In addition, we also introduced the source, chemical structure, chemical properties, and toxicity of DHM in this review. We aim to deepen our understanding of DHM on inflammation-related diseases, clarify the relevant molecular mechanisms, and find out the problems and solutions that need to be solved urgently. Providing new ideas for DHM drug research and development, as well as broaden the horizons of clinical treatment of inflammation-related diseases in this review. Moreover, the failure of clinical transformation of DHM poses a great challenge for DHM as an inflammation related disease.

## Introduction

Dihydromyricetin (DHM) is a flavonoid mainly derived from Nekemias grossedentata (Hand.-Mazz.) J.Wen and Z.L.Nie (*N.grossedentata*) (Liu et al., 2019a). Flavonoids have multifarious pharmacological effects, such as antioxidant, anti-inflammatory response, anti-cancer, and anti-viral as well as neuroprotective effects (Wang et al., 2016a). DHM exists not only in *N. grossedentata*, but also in plant food (Wu et al., 2015). There are many inflammatory diseases that affect peoples’ physical and mental health as well as the quality of life. For instance, atherosclerosis (Geovanini and Libby, 2018), diabetic cardiomyopathy (Li et al., 2019), endothelial dysfunction (Bai et al., 2020), neurodegenerative diseases (Stephenson et al., 2018), and cancer (Diakos et al., 2014) as well as liver disease (Yang et al., 2019), and so forth. In addition, DHM has numerous biological effects, including anti-oxidation, improving mitochondrial dysfunction, and regulating autophagy (Liu et al., 2020), especially anti-inflammatory effects (Zhang et al., 2018). This suggests that DHM exerts its pharmacological effects through the corresponding molecular mechanisms.

## N. grossedentata

*N. grossedentata* has been used as an herbal medicine belonging to the *Parthenocissus inserta f. dubia* (Rehder) Rehder J. Arnold Arbor (Ma et al., 2020) in China for thousands of years. “Compendium of Vatica rassak (Korth.) Blume Medica” believed that it had the effect of “regulating the middle and replenishing qi, promoting blood and promoting qi” (Hu et al., 2020), which was used as tea (Zhou et al., 2014). It is generally known as that Calamus rotang L. tea is used as a heat clearing-herb in traditional Chinese medicine to promote diuresis and blood circulation (Huang et al., 2018). In addition, *N. grossedentata* is common in South China and can also be eaten (Jia et al., 2020). The leaves (Li et al., 2020) and stems (Gao et al., 2017) of the *N. grossedentata* are also known as vine tea. Its tender stems and leaves are widely used as *Citrullus colocynthis* (L.) Schrad. tea. It has been used for herbal tea and traditional Chinese medicine for hundreds of years. Modern pharmacological studies have shown that *N. grossedentata* has a variety of pharmacological effects, including antioxidative, anti-inflammatory, and antiviral (Chen et al., 2016) as well as antithrombotic (Sun et al., 2020). Therefore, *N. grossedentata* treated many diseases clinically, such as diabetes (Chen et al., 2016), pharyngitis, sore throat, and fever associated with colds (Hou et al., 2015a).

## Dihydromyricetin

**FIGURE 1:**
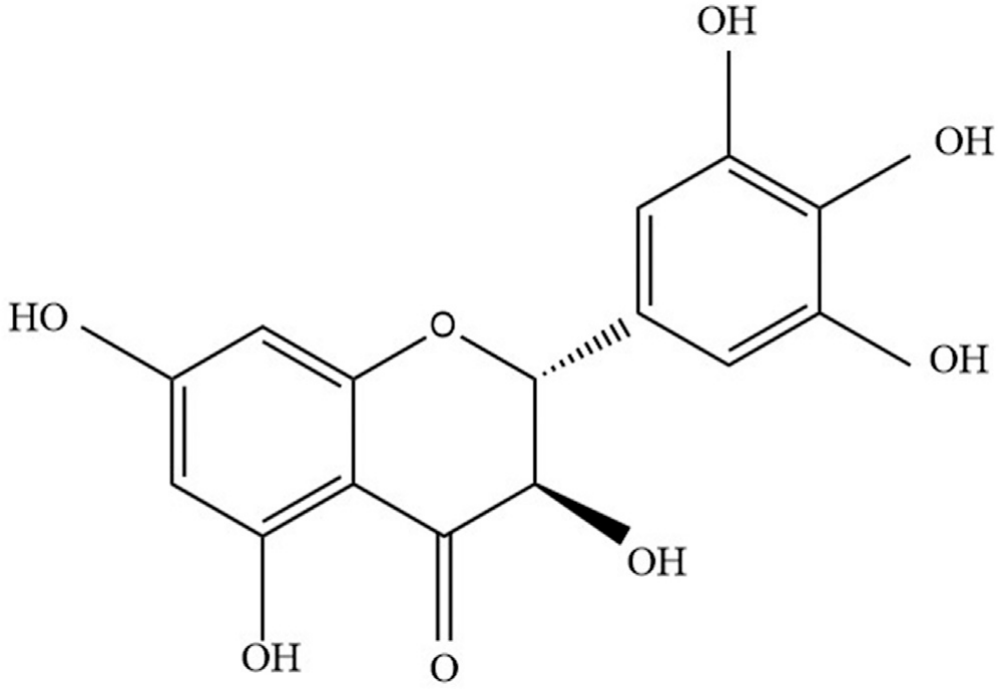
The chemical structure of DHM.

DHM (3′, 4′, 5, 5′, 7-Hexahydroxy-2, 3-dihydroflavanonol) was first discovered from *Nekemias meliaefolia* in 1940 (Zhang et al., 2001) (Figure 1). Therefore, DHM is also known as *Ampelopsis japonica (Thunb)* Makino (AMP) (Liu et al., 2020). The content of DHM in *N. grossedentata* was as high as 30–40% (Liu et al., 2019a). DHM was also found in *Vitis vinifera L., Myrica cerifera L., Prunus amygdalus Batsch, Ginkgo biloba L*., and other plants (Liu et al., 2020). In addition, studies have confirmed the existence of DHM in Hovenia dulcis Thunb and Cedrus deodara (Roxb. ex D.Don) G.Don (Liang et al., 2014a; Liang et al., 2014b). DHM will degrade in a weak alkaline environment, especially under the condition of Cu^2+^ and Fe^3+ 25^. DHM was poorly soluble, only soluble in hot water and ethanol (Liu et al., 2019b). The efficacy of DHM will be affected by its bioavailability (Feng et al., 2018). The animal experimental data showed that the bioavailability of DHM in rats was 4.02% (Liu et al., 2017a). The main metabolic sites of DHM are in the liver and gastrointestinal tract, and some are absorbed by the blood. Following being absorbed by the blood, DHM can be distributed throughout the body (Ding et al., 2021). DHM was characterized by low bioavailability and unstable chemical properties, which limited the pharmacology and clinical application of DHM (Tong et al., 2015). And for DHM, only a few kinds of studies can conclude that DHM is not toxic (Zhang et al., 2014).

## Inflammation and Inflammatory Diseases

Following sterile tissue injury, the positive response of the host to pathogens is called “inflammation” (Alessandri et al., 2013). Inflammation is divided into acute inflammation and chronic inflammation (Arulselvan et al., 2016). When inflammation is triggered, it leads to the recruitment and activation of neutrophils, monocytes, macrophages, and other immune cells (He et al., 2020). Macrophages are the first immune cells affected by the inflammatory response (Lopez-Castejon, 2020). In addition, the inflammatory process is regulated by cytokines. Cytokines are secreted by immune cells (Kirkpatrick and Miller, 2013). Inflammation is a multi-stage and complex process, involving a variety of cells as well as signal cascades (Cas et al., 2020). The types of inflammation include acute inflammation and chronic inflammation (Arulselvan et al., 2016). In recent years, inflammation has been a research hotspot. Studies have shown that there is a complex relationship between inflammation and inflammatory disease. For example, atherosclerosis, heart failure (Shirazi et al., 2017), rheumatoid arthritis (Dhingra and Chopra, 2020), neurodegenerative diseases (Baune, 2015), cancer (Sgambato and Cittadini, 2010), and cardiac arrhythmogenesis (Yalta and Yalta, 2018). In general, many diseases are associated with inflammation, so we must pay attention to it.

## Anti-inflammatory Mechanism of DHM

### NLRP-3 and Pyroptosis

Some studies have confirmed that DHM is closely related to palmitic acid (PA)-induced human umbilical vein endothelial cells (HUVECs). Hu et al. have clarified that DHM ameliorated pyroptosis by activating the Nrf2 (NF-E2-related factor 2) signaling pathway. DHM inhibited the activation of NLRP-3 by down-regulating mitochondrial reactive oxygen species (ROS) in PA-induced HUVECs (Hu et al., 2018). DHM treatment inhibited the activation of caspase-1 and the expression of IL-1β in the brain of AD mice. This suggested that DHM inhibited the activation of the NLRP-3 signaling pathway to improve the inflammatory response in AD (Liu et al., 2019b). In addition, DHM improved pyroptosis caused by NLRP-3 in chronic liver injury mice (Cheng et al., 2020). DHM significantly inhibited cholesterol accumulation and foam cell formation, improved mitochondrial function, reduced oxidative stress as well as reduced the activation of NLRP3 in oxidized low-density lipoprotein (ox-LDL)-stimulated macrophages in Sirtuin3 (SIRT3) ko mice (Sun et al., 2021). Studies have shown that NLRP-3 activation contributed to the development of cardiotoxicity. What is more, DHM improved myocardial injury by inhibiting the activation of NLRP-3 ^20^.

### Nuclear Factor-κB (NF-κB)

Research confirmed that DHM down-regulated the expression of the NF-κB signal pathway by directly binding to IκB kinase (IKK), thereby inhibiting IKK phosphorylation. To sum up, DHM exerted an antiarthritic effect in collagen-induced arthritis rats through down-regulation of NF-κB (Wu et al., 2019a). Tang et al. confirmed by Western blotting that DHM inhibited tumor necrosis factor-α (TNF-α)-induced phosphorylation of IKKα/β dose-dependently. The results suggested that DHM significantly inhibited the expression of NF-κB, and then inhibited the inflammatory response (Tang et al., 2016). DHM inhibited the activation of macrophage by suppressing NF-κB p65 phosphorylation, IKKβ activity, and IKKα/β phosphorylation in the NF-κB pathway (Wang et al., 2016b). Similarly, the research results of Wu et al. showed that DHM inhibited the activation of the NF-κB pathway by attenuating the phosphorylation of the NF-κB in rheumatoid arthritis (Wu et al., 2020).

### Cytokines

Liu et al. have confirmed that DHM improved the inflammatory response of the liver and aorta by inhibiting the expression of TNF-α as well as Interleukin (IL)-6 in LDL receptor-deficient mice (Liu et al., 2017b). DHM reduced oxidative stress and down-regulated the levels of TNF-α, IL-6, IL-1β, and cyclooxygenase-2 (COX-2) by activating Nrf2 in rheumatoid arthritis rat (Chu et al., 2018). Studies have confirmed that there is a close relationship between inflammation and the liver damage. DHM prevented TNF-α mediated liver toxicity by inhibiting the expression of TNF-α through the c-Jun N-terminal kinase (JNK) signaling pathway (Xie et al., 2015). Wu et al. have explained that the levels of IL-6 and TNF-α in diabetic cardiomyopathy mice treated with DHM were significantly reduced (Wu et al., 2017). In addition, DHM inhibited the expression of inflammatory factors (IL-6 and TNF-α) in rats with pulmonary hypertension (PH) (Li et al., 2017). DHM inhibited the expression of IL-4, IL-5, and IL-13 in alveolar lavage fluid in asthmatic mice (Xu et al., 2017).

### Neuroinflammation

**FIGURE 2:**
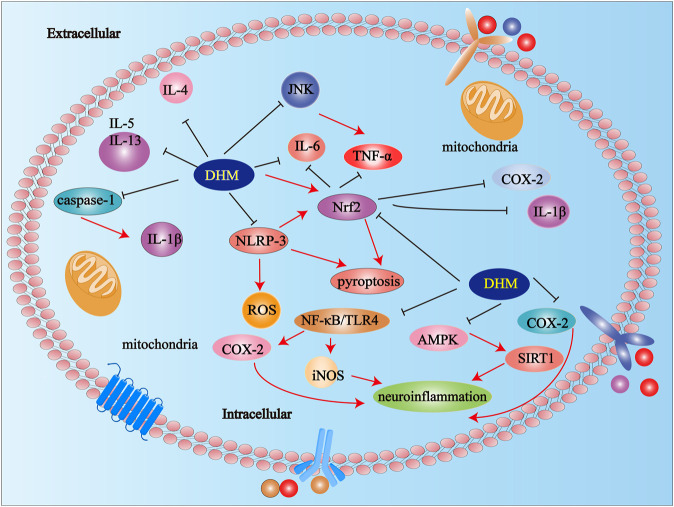
Anti-inflammatory mechanisms of DHM. Abbreviations: IL-5, Interleukin-5; IL-13, Interleukin-13; IL-4, Interleukin-4; DHM, Dihydromyricetin; Interleukin-1β; Nrf2, NF-E2-related factor 2; TNF-α, tumor necrosis factor; COX-2, cyclooxygenase-2; JNK, c-Jun N-terminal kinase; iNOS, inducible nitric oxide synthase; STAT, signal transducer and activator of transcription; NF-κB, Nuclear factor-κB.

Neuroinflammation plays a significant role in many neurological diseases, such as depression (Troubat et al., 2021), AD (Calsolaro and Edison, 2016), stroke (Jayaraj et al., 2019), Parkinson’s disease (PD) (Kustrimovic et al., 2019), amyotrophic lateral sclerosis (ALS) (Liu and Wang, 2017), and so on. DHM down-regulated the level of cytokines via NLRP-3 signaling pathway in AD mice (Liu et al., 2019a). Studies have confirmed that DHM effectively improved astrocyte and microglia mediated neuroinflammation (Wu et al., 2019b). DHM inhibited neuroinflammation in AD rats through adenosine 5′-monophosphate activated protein (AMPK)/signal transducer and activator of transcription 1 (SIRT1) pathway (Liu et al., 2020). DHM improved depressive symptoms by alleviating neuroinflammatory response (Ren et al., 2018). DHM inhibited the inflammatory response, inhibited the secretion of inducible nitric oxide synthase (iNOS) and COX-2, and attenuated the activation of NF-κB and TLR4 signals in lipopolysaccharide (LPS)-induced neuroinflammation (Jing and Li, 2019). DHM inhibited inflammatory responses via up-regulation of the AMPK/SIRT1 pathway in AD mice (Sun et al., 2019) (Figure 2).

## Other Mechanisms of DHM Except for Anti-inflammatory

**FIGURE 3:**
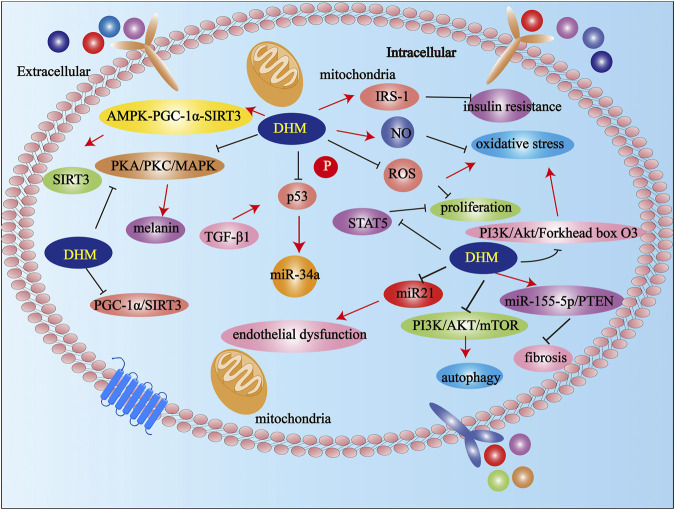
Possible mechanisms of DHM in others expect anti-inflammation. Abbreviations: DHM, Dihydromyricetin; NO, nitric oxide; AKT, protein kinase B; PI3K, Phosphatidylinositol 3-kinase; PGC-1α, peroxisome proliferator-activated receptor γ coactiva-tor-1; IRS-1, ginsulin receptor substrate-1; miR-34a, microRNA-34a; ROS, reactive oxygen species.

Furthermore, DHM reduced inflammatory response via the JNK pathway in acute liver injury mice (Wang et al., 2018a). DHM reversed the metabolic syndrome by upregulating insulin receptor substrate-1 (IRS-1) (y612) tyrosine phosphorylation and improving insulin resistance in obese mice (He et al., 2019). Of note, DHM inhibited the production of melanin by down-regulating the protein kinase A (PKA), protein kinase C (PKC), and mitogen-activated protein kinases (MAPK) signaling pathways in B16F10 cells (Huang et al., 2016). DHM inhibited the growth of *Staphylococcus aureus* by destroying the integrity of the cell wall and increasing the permeability of the cell membrane (Liang et al., 2020). Studies have shown that DHM promoted SIRT3 in chondrocytes via the AMPK-peroxisome proliferator-activated receptor γ coactiva-tor-1 (PGC-1α)-SIRT3 signaling pathway in osteoarthritis rat (Wang et al., 2018b). DHM significantly reversed cisplatin-induced nephrotoxicity by reducing oxidative stress and inhibiting apoptosis (Wu et al., 2016). Studies have shown that DHM down-regulated microRNA-34a (miR-34a) in renal tubular epithelial cells by inhibiting the phosphorylation of p53 (tumor suppressor gene) induced by transforming growth factor β1 (TGF-β1) (Liu et al., 2019c). In addition, DHM ameliorated memory impairment caused by DHM and improves memory impairment caused by hypobaric hypoxia (Liu et al., 2016). DHM improved oxidative stress by inhibiting ROS production and increasing nitric oxide (NO) production in endothelial cells (Hou et al., 2015b). DHM reduced the production of inflammatory factors in mast cells by inhibiting signal transducer and activator of transcription 5 (STAT5) and the NF-κB signaling pathways. DHM improved mast cell proliferation by significantly attenuating IgE-induced ROS and inhibiting STAT5 phosphorylation in mast cells (Chang et al., 2021). DHM improved emodin-induced hepatotoxicity by inhibiting oxidative stress via the Nrf2 signal pathway in human hepatocyte cell line L02 ^74^. Studies have confirmed that DHM improved gentamicin-induced ototoxicity through the PGC-1α/SIRT3 signaling pathway in house ear institute-organ of corti (HEI-OC)1 (Han et al., 2020). DHM promoted autophagy and improved renal interstitial fibrosis in diabetic nephropathy (DN) by regulating the miR-155-5p/phosphatase and tensin homolog deleted on chromosome ten (PTEN) signaling pathway and phosphatidylinositol 3-kinase (PI3K)/protein kinase B (AKT)/mammalian target of rapamycin (mTOR) signaling pathway (Guo et al., 2020a). Studies have shown that DHM improved TNF-α-induced endothelial dysfunction by inhibiting miR-21 ^77^. DHM improved oxidative stress in endothelial cells through PI3K/Akt/Forkhead box O3 (FoxO3a) pathway (Zhang et al., 2019). DHM significantly changed the richness and diversity of the intestinal microbiota and regulated the composition of the intestinal microbiota (Fan et al., 2018). Studies have shown that long-term use of DHM attenuated the development of PD-like behaviors and pathological phenotypes (Guo et al., 2020b). In addition, DHM is also a new type of anti-alcoholism drug (Shen et al., 2012). DHM improved liver function and brain histopathology in mice with acute liver failure related the hepatic encephalopathy (Cheng et al., 2021). In addition, DHM also has anti-thrombotic effects (Chen et al., 2021a) (Figure 3).

## Possible Crosstalk Between Anti-inflammatory and Other Effects in DHM

DHM improved TNF-α-induced endothelial dysfunction by inhibiting miR-21 (Yang et al., 2018). We speculated whether DHM inhibited a series of inflammatory responses induced by TNF-α by inhibiting the expression of miR-21. DHM inhibited inflammatory response via up-regulation of the AMPK/SIRT1 pathway in AD mice (Sun et al., 2019). DHM inhibited neuroinflammation in AD rats through AMPK/SIRT1 pathway (Liu et al., 2020). This suggested that in addition to the anti-inflammatory pathway, DHM mediated other pathways to play an anti-inflammatory role in inflammatory diseases. DHM exerted other anti-inflammatory effects through anti-inflammation. On the contrary, DHM exerted anti-inflammatory effects via other related signaling pathways. This is an exciting and interesting crosstalk, which is worthy of in-depth exploration and discovery in future research. We can knock out an inflammatory gene by gene knockdown and observe the changes of other pathways after DHM treatment. Or more attention should be paid to other related pathways in the study of inflammatory diseases in DHM.

## Clinical Study of Dihydromyricetin

We consulted the studies on DHM in the most recent 20 years, and we found that the clinical reports on DHM were very limited. There were few studies about the anti-inflammatory effect of DHM in the clinic. Sixty adult patients with nonalcoholic fatty liver disease underwent a randomized double-blind experiment. Following DHM treatment, the level of TNF-α and cytokeratin-18 in serum decreased significantly in this group (Chen et al., 2021b). In a randomized double-blind trial of 80 patients with type two diabetes for 1 month, data showed that DHM supplementation significantly improved renal function parameters and glycemic control in patients with type two diabetes mellitus (Ran et al., 2019). We should pay more attention to DHM in clinical practice. We should explore the mechanism of DHM through animal and cell experiments to solve the unsolved problems in the clinic.

## Challenges and Difficulties of Dihydromyricetin in Clinic

Studies have shown that DHM is only 0.2 mg/ml at 25°C, the solubility of DHM is very low, so DHM cannot be completely absorbed from the intestine (Ran et al., 2019). Xiang et al. evaluated the passive diffusion absorption capacity of DHM through human internal Caco-2 cells. The results showed that the passive diffusion absorption capacity of DHM was very poor, and the uptake and transport of DMY depended on time and concentration. PH value affects DMY uptake but not its two-way transport (Xiang et al., 2018). Because of its poor bioavailability and absorption capacity, its clinical application is limited (Ran et al., 2019). Thus far, many studies have reported that DHM inhibited the proliferation of many types of human tumor cells, such as human cholangiocarcinoma cells (Chen et al., 2020), human intestinal Caco-2 cells (Xiang et al., 2018), human ovarian cancer cells (Wang et al., 2019), and human myelomonocytic lymphoma cells (Feng et al., 2019), and so on. However, there are few studies on anti-tumor or else pharmacological effects *in vivo* or clinic. To better apply DHM in the clinic, improving the bioavailability and gastrointestinal absorption of DHM is not only an urgent problem to be solved but also difficult and challenging for the clinical application of DHM.

## Discussion

**FIGURE 4:**
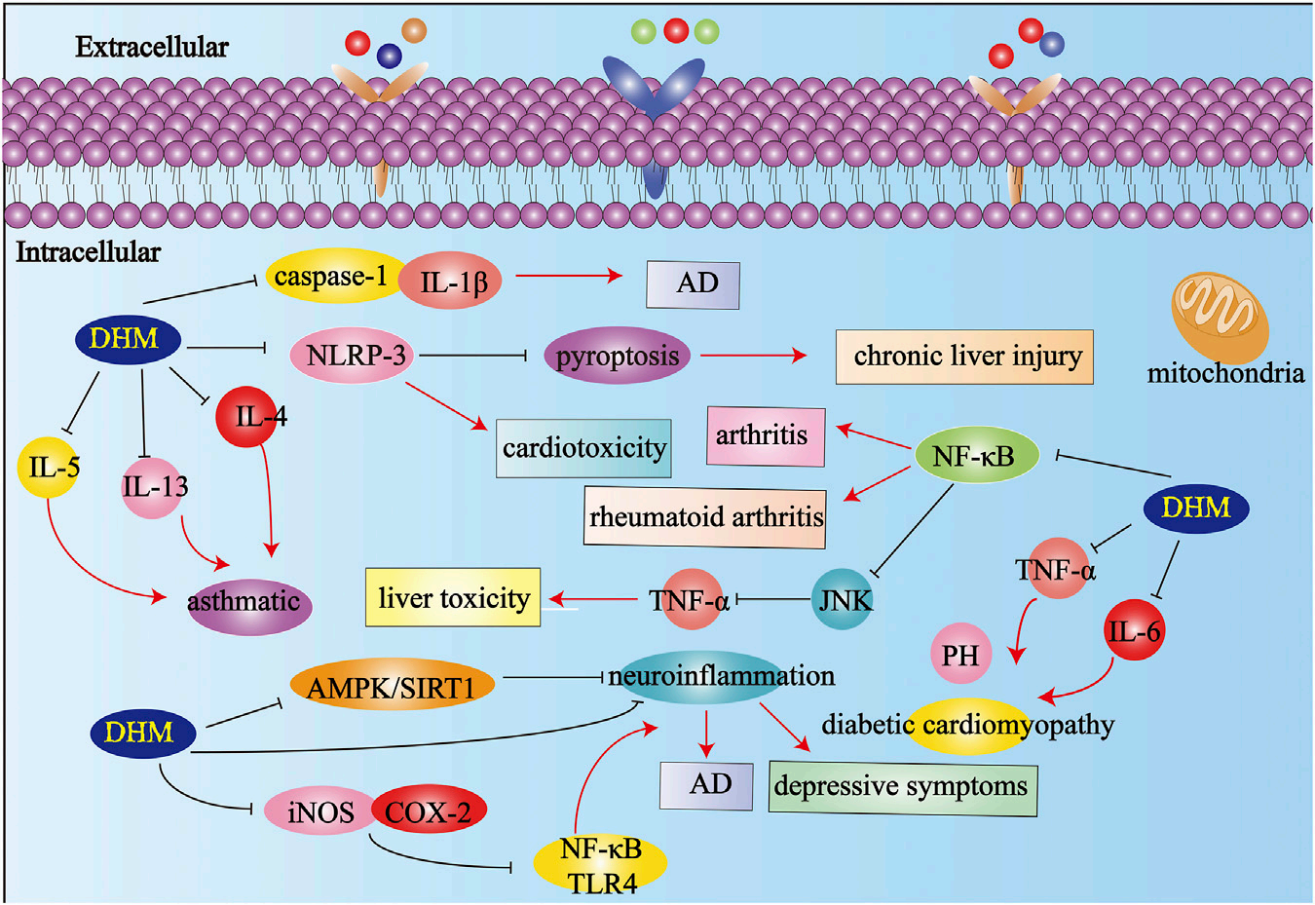
The effects and mechanisms on different inflammatory diseases. Abbreviations: AD, Alzheimer’s disease; PH, pulmonary hypertension; IL-1β, Interleukin-1β; IL-5, Interleukin-5; IL-13, Interleukin-13; IL-4, Interleukin-4; IL-6, Interleukin-6; iNOS, inducible nitric oxide synthase; TNF-α, tumor necrosis factor-α; COX-2, cyclooxygenase-2; JNK, c-Jun N-terminal kinase; AMPK, Adenosine 5′-monophosphate activated protein; NF-κB, Nuclear factor-κB; DHM, Dihydromyricetin; SIRT, Sirtuin.

Numerous studies have shown that inflammation plays a vital role in a variety of diseases, including diabetes, arthritis, asthma (Zhong and Shi, 2019), AD, acute cerebral stroke, cancer, hypertension, and myocardial ischemia (Liu et al., 2019a; Figure 4). Therefore, the further elucidation of inflammation-related mechanisms and the development of anti-inflammatory drugs are urgent problems to be solved. DHM has been used to treat different diseases for a long time (Martínez-Coria et al., 2019). This review summarizes the mechanism of DHM in inflammatory diseases according to its different effects systematically, focusing on the research progress of DHM in anti-inflammatory, apoptosis, oxidative stress, and the effects of various metabolic pathways. In summary, many research results show that DHM as a component of natural medicine has a variety of pharmacological effects. As we all know, numerous diseases are closely related to inflammation (Medzhitov, 2008). Therefore, we should pay more attention to the research and development of anti-inflammatory mechanisms and anti-inflammatory drugs. DHM has multiple pharmacological effects, especially anti-inflammatory pharmacological effects. However, the specific mechanism and many targets of DHM’s anti-inflammatory pharmacological effects need to be further studied and explored. Although a large amount of literature has prompted us, DHM is a potential drug for the treatment of inflammation-related diseases (Table 1). Thus far, most studies on DHM have focused on animal and cell levels, and it is not common for clinical studies. Although studies have confirmed that DHM can inhibit the proliferation of a variety of human tumor cells, it is still limited to the cell level. This is a problem that demands us to think and solve. In future research, we should pay attention to the clinical transformation of DHM. The bioavailability and chemical stability of DHM can be improved by changing the dosage form into the sustained-release mechanism, controlled-release preparation, or targeted preparation. DHM is an active compound mainly derived from *A.grossedentata.* It can also be combined with other related drugs to make compound preparations to improve the curative effect and bioavailability.

**TABLE 1:** The summary of mechanisms of DHM. The latest research progress and related mechanisms of DHM in animals, cells, and clinic were summarized

Experimental model/patients	Mechanism	References
AD mice	Down-regulation of caspase-1, IL-1β, and NLRP-3 expression	Feng et al. (2018)
Chronic liver injury mice	Down-regulation of NLRP-3 expression	Cheng et al. (2020)
SIRT3 ko mice	Down-regulation of NLRP-3 and oxidative stress	Liu et al. (2019b)
Myocardial injury rat	Down-regulation of NLRP-3 expression	Sun et al. (2020)
Arthritis rats	Down-regulation of NF-κB expression	Wu et al. (2019a)
Rheumatoid arthritis rat	Down-regulation of P-NF-κB expression	Wu et al. (2020)
LDL receptor-deficient mice	Down-regulation of TNF-α and IL-6 expression	Liu et al. (2017b)
Rheumatoid arthritis rat	Down-regulation of TNF-α, IL-6, IL-1β, and COX-2 expression	Chu et al. (2018)
	Up-regulation of Nrf2 expression	
Liver damage mice	Down-regulation of TNF-α and JNK expression	Xie et al. (2015)
Diabetic cardiomyopathy mice	Down-regulation of TNF-α and IL-6 expression	Wu et al. (2017)
Pulmonary hypertension rat	Down-regulation of TNF-α and IL-6 expression	Li et al. (2017)
Asthmatic mice	Down-regulation of IL-4, IL-5, and IL-13 expression	Xu et al. (2017)
AD rat	Inhibition of AMPK/SIRT1 signal pathway	(Liu et al., 2020), (Sun et al., 2019)
Acute liver injury mice	Down-regulation of JNK expression	Wang et al. (2018a)
Obese mice	Up-regulation of IRS-1 expression	He et al. (2019)
Osteoarthritis rat	Down-regulation of SIRT3 expression	Wang et al. (2018b)
HUVECs	Up-regulation of Nrf2 expression	Hu et al. (2018)
	Down-regulation of NLRP-3 and ROS expression	
B16F10 cells	Down-regulation of PKA, PKC, MAPK, and melanin expression	Huang et al. (2016)
Tubular Epithelial Cells	Down-regulation of miR-34a expression	Liu et al. (2019c)
Endothelial cells	Down-regulation of ROS expression	Hou et al. (2015b)
	Up-regulation of NO expression	
Mast cell	Down-regulation of STAT5 and NF-κB expression	Chang et al. (2021)
L02 cell	Up-regulation of Nrf2 expression	Yan et al. (2019)
Endothelial cells	Inhibition of PI3K/Akt/FoxO3a signal pathway	Guo et al. (2020a)
Patients with renal fibrosis	Down-regulation of miR-34a expression	Liu et al. (2019c)
Patients with nonalcoholic fatty liver	Down-regulation of TNF-α and cytokeratin-18 expression	Chen et al. (2021b)
patients with type two diabetes mellitus	Down-regulation of glycemic expression	Ran et al. (2019)

Abbreviations: AD, Alzheimer’s disease; COX-2, cyclooxygenase-2; House Ear Institute-Organ of Corti, HEI-OC; HUVECs, human umbilical vein endothelial cells; IL-1β, Interleukin-1β; IL-13, Interleukin-13; IL-4, Interleukin-4; IL-5, Interleukin-5; IL-6, Interleukin-6; IRS-1, ginsulin receptor substrate-1; JNK, c-Jun N-terminal kinase; LDL, low-density lipoprotein; L02, human hepatocyte cell; miR-34a, microRNA-34a; NF-κB, Nuclear factor-κB; NO, nitric oxide; Nrf2, NF-E2-related factor 2; PGC-1α, peroxisome proliferator-activated receptor γ coactiva-tor-1; P-NF-κB, Phosphorylation-nuclear factor-κB; STAT5, signal transducer and activator of transcription 5; ROS, reactive oxygen species; Sirtuin3, SIRT3; TNF-α, tumor necrosis factor-α

**FIGURE 5:**
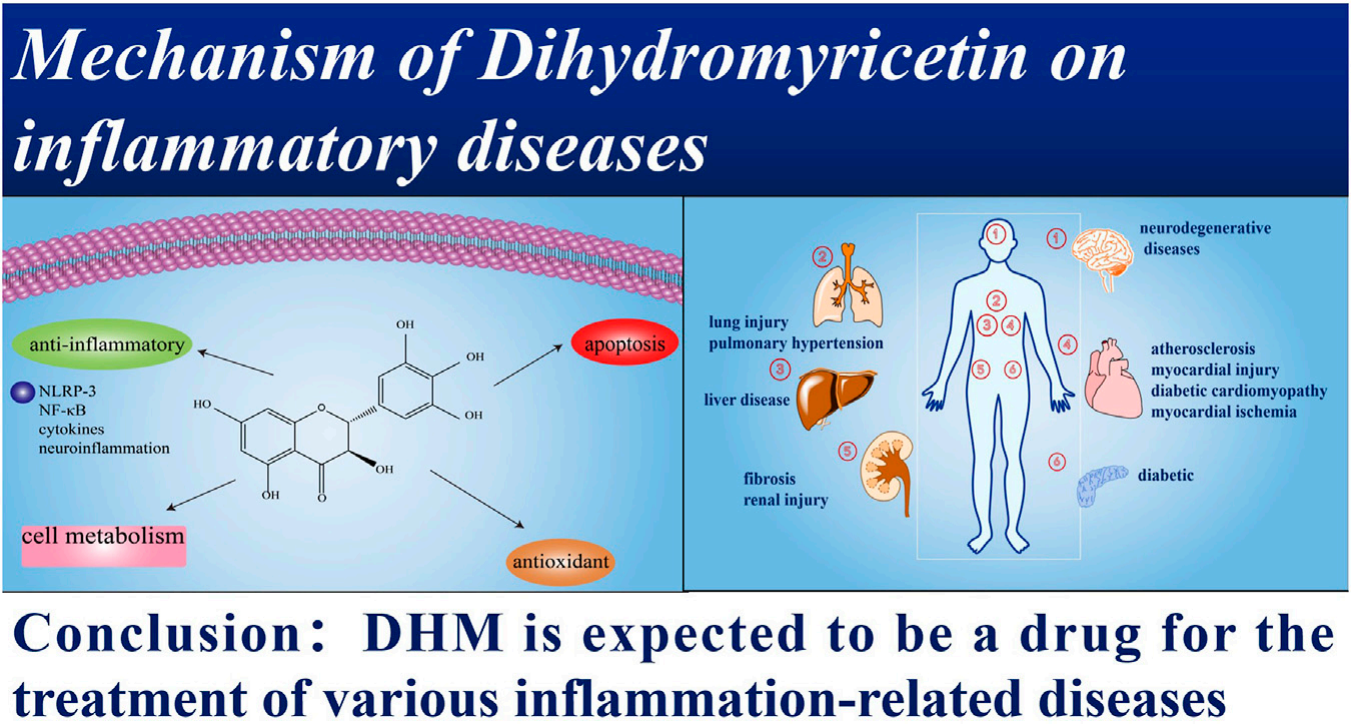
DHM is expected to be a drug for the treatment of various inflammation-related diseases.

The clinical transformation of candidate drugs or active compounds is an urgent problem to be solved in future research, which is meaningful and valuable life science research (Liu et al., 2019d; Figure 5).
